# Prevalence, patterns and associated risk factors for dyslipidaemia among individuals attending the diabetes clinic at a tertiary hospital in Central Malawi

**DOI:** 10.21203/rs.3.rs-3262287/v1

**Published:** 2023-08-21

**Authors:** Florence Filisa-Kaphamtengo, Jonathan Ngoma, Victoria Mukhula, Zaithwa Matemvu, Deborah Kapute, Peter Banda, Tamara Phiri, Mwapatsa Mipando, Mina C. Hosseinipour, Kondwani G.H. Katundu

**Affiliations:** Kamuzu Central Hospital; Kamuzu Central Hospital; Malawi-Liverpool-Wellcome Trust Clinical Research Programme; Kamuzu Central Hospital; Blantyre Adventist Hospital; Kamuzu University of Health Sciences; Queen Elizabeth Central Hospital; Kamuzu University of Health Sciences; The University of North Carolina Project-Lilongwe; Kamuzu University of Health Sciences

**Keywords:** Dyslipidaemia, Atherosclerotic cardiovascular disease, Diabetes mellitus, Kamuzu Central Hospital, Malawi

## Abstract

**Background:**

Dyslipidaemia among individuals with diabetes is a significant modifiable risk factor for atherosclerotic cardiovascular diseases (ASCVDs). ASCVDs are a major cause of mortality and morbidity globally, especially in people with diabetes. In Malawi, limited data exist on the prevalence and biochemical characteristics of diabetic dyslipidaemia. This study investigated the prevalence and biochemical characteristics of dyslipidaemia in individuals attending the diabetes clinic at Kamuzu Central Hospital, the largest tertiary referral hospital in Central Malawi.

**Methods:**

Using a cross-sectional design, sociodemographic, medical and anthropometric data were collected from 391 adult participants who were enrolled in the study. Blood samples were analysed for glycosylated haemoglobin (HBA1c) and fasting lipid profiles. The prevalence of dyslipidaemia was calculated, and the biochemical characteristics of the dyslipidaemia were defined. The associations between dyslipidaemia and risk factors such as sociodemographic characteristics, obesity, and HBA1c levels were evaluated using logistic regression analysis.

**Results:**

Prevalence of dyslipidaemia was observed in 71% of the participants, and elevated low-density lipoprotein cholesterol was the most frequent lipid abnormality among the study participants. On bivariate analysis, dyslipidemia was positively associated with female sex [OR 1.65 (95% CI 1.05–2.58); p = 0.09], age ≥ 30 years [OR 3.60 (95% CI 1.17–7.68); p = 0.001] and overweight and obesity [OR 2.11 (95% CI 1.33–3.34); p = 0.002]. On multivariate analysis, being overweight or obese was an independent predictor of dyslipidaemia [AOR 1.8 ;( 95% CI 1.15–3.37); p = 0.04].

**Conclusion:**

Dyslipidaemia was highly prevalent among individuals with diabetes in this study, and elevated low-density lipoprotein cholesterol was the most frequent lipid abnormality. Overweight and obesity were also highly prevalent and positively predicted dyslipidaemia. This study highlights the importance of appropriately addressing dyslipidaemia, overweight and obesity among individuals with diabetes in Malawi and other similar settings in Africa as one of the significant ways of reducing the risk of ASCVDs among this population.

## Background

Approximately 537 million adults are living with diabetes mellitus (DM) globally, 75% of which live in low and middle-income countries (LMICs) (1). In Malawi, the prevalence of DM is estimated at 7% (2). DM is a significant risk for atherosclerotic cardiovascular diseases (ASCVDs), such as stroke, which is the major cause of morbidity and mortality in people living with DM (3). ASCVDs cause 31% of all global deaths, with 80% occurring in LMIC (4). Dyslipidaemia complicates DM and doubles the risk of cardiovascular events in individuals with DM (3, 5). Dyslipidaemia is a driver of atherosclerosis and is an indirect cause of at least 2 million annual deaths and nearly 30 million disabilities globally (4).

Dyslipidaemia denotes a group of lipid abnormalities characterised by one or more of the following: elevated total cholesterol (TC), elevated low-density lipoprotein cholesterol (LDL-C), decreased high-density lipoprotein cholesterol (HDL-C), and elevated triglycerides (TG) (4, 6). Diabetic dyslipidaemia is typically characterised by elevated triglycerides (TG), decreased high-density lipoprotein cholesterol (HDL-C) with normal to mildly elevated low-density lipoprotein cholesterol (LDL-C), owing to the overproduction of TG-rich very-low-density lipoprotein (VLDL) particles in the liver and increased exchange of TG in VLDL for cholesteryl esters in HDL and LDL-producing sdLDL (7-9).

There is limited data on the prevalence, biochemical characteristics, and risk factors of dyslipidaemia among individuals with DM in Malawi. This cross-sectional study aimed to determine the prevalence of dyslipidaemia, biochemical characteristics, and the associated risk factors among individuals with DM attending the adult DM clinic at Kamuzu Central Hospital (KCH), the largest tertiary referral hospital in Central Malawi.

## Methods

### Study design and setting

This quantitative cross-sectional study was conducted at the adult KCH, the largest tertiary referral hospital in Lilongwe, in central Malawi. KCH serves a population of about 7 million people in Central Malawi. The study population was adults aged 18 years and above with DM, either type 1 (T1DM) or type 2 (T2DM), irrespective of HIV status and attending the KCH DM clinic. Data were collected between March and June 2021.

### Inclusion and exclusion criteria

Participants were included in the study if they were confirmed DM patients aged 18 years and older and consented to participate. We excluded pregnant participants, those with an incomplete medical history, those with fever or history of an active infection, and those from whom blood sample collection was unsuccessful.

### Study population and sampling strategy

Study participants were enrolled when they visited the DM clinic. The study period coincided with the Coronavirus disease 2019 (COVID-19) pandemic and there were limited booked patients for the clinics. In that case, consecutive sampling was used to recruit participants in the study until the sample size was reached.

The clinical research nurse collected and documented data using an interviewer-administered questionnaire. Sociodemographic data were collected and recorded. Weight and height measurements were performed and recorded, from which the body mass index (BMI) was calculated. Clinical nurses also collected two blood samples in ethylenediaminetetraacetic acid (EDTA) tube for HBA1c tests and another in a plain tube for fasting serum lipid profile testing for LDL-C, TG, TC and HDL using an automated Erba XL640 (USA) by a qualified laboratory technologist.

### Definition of Parameters

Dyslipidaemia was defined as the presence of one or more lipid abnormalities among the study participants: TC > 200mg/dl, LDL-C > 100mg/dl, TG > 150 mg/dl and HDL-C < 40 mg/dl (10, 11). Dyslipidaemia was further classified as isolated when a single abnormal lipid parameter (TC, TG, HDL-C or LDL-C) was present; combined when two lipid parameters (elevated TG, low HDL-C or elevated LDL-C) were detected; and mixed when all three lipid parameters are abnormal (elevated TG, low HDL-C and elevated LDL-C) (3). Poor glycaemic control was defined as HBA1c of > 7% (10). BMI categories were classified according to the WHO classification as underweight if BMI < 18.5, normal if BMI was between 18.5 and 25, overweight if BMI was between ≥ 25.0 and 30, and obese if BMI ≥ 30.0 (12).

### Statistical analysis

Data were entered Microsoft Excel spreadsheet and statistical analysis was performed using STATA 17 (StataCorp LLC). The Shapiro- Wilks normality test was used to test the data for normality. Descriptive statistics for continuous data were expressed as the means or medians, and proportions for categorical data. The Chi-squared or Fisher's exact test for independent variables was used to compare categorical data. The t-test was used to analyse the differences in mean difference in lipid concentrations between any two groups. Bivariate logistic regression was used to evaluate risk factors for dyslipidaemia, such as age groups, DM type, sex, or BMI categories. Multivariate logistic regression analysis accounted for confounding and included all variables with a p < 0.1 on bivariate analysis. In all cases, a *p*-value < 0.05 was considered significant.

## Results

### Participants Recruitment Process

**Figure 1:**
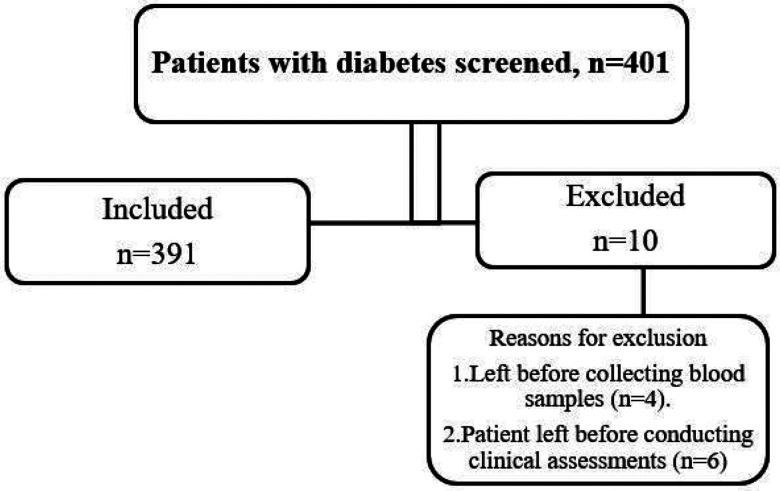
Strobe diagram illustrating the participant recruitment process, number of participants screened, inclusion and the reasons for exclusion in the study

A total of 401 participants were screened for inclusion in the study, and 391 were enrolled (Figure 1). Participants were excluded from the study because they left before conducting clinical assessments or blood sample collection was not performed before blood pressure measurement (Figure 1).

### Participant's sociodemographic and clinical characteristics

Most of the study participants were females (64%), middle-aged with a mean age of 52 (13) years (Table 1). The HIV prevalence in the population was 11%, but the status was unknown in 13% of the participants. The mean blood pressure readings were 142 (26) mmHg systolic and 87.50 (15) mmHg diastolic. The mean HbA1c level was 10.29 (3.35).

**Figure 2:**
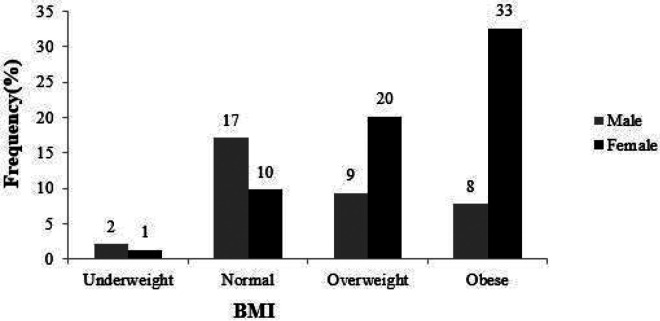
Body Mass index categories by sex

The prevalence of overweight and obesity in the study was 70% (30% overweight and 40% obese, respectively). Figure 2 shows the BMI categories classified by sex. Female participants were more likely to be overweight and obese than the male participants [OR 5.32 (95% CI, 3.34 - 8.47); p<0.001].

### Prevalence of dyslipidaemia and the biochemical patterns in the study population

**Figure 3:**
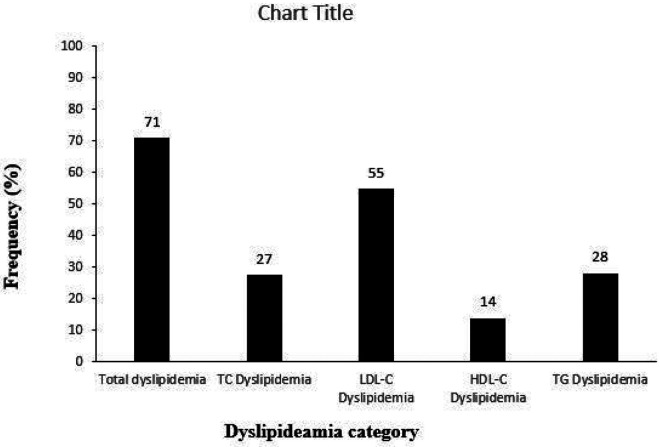
Frequency of lipid abnormalities in the study participants. Total dyslipidemia represents all individuals with dyslipidemia in study population. Elevated LDL-C dyslipidaemia was the most common lipid abnormality observed. The sum of the frequencies exceed 100% because most of the participants had a combination of at least two lipid abnormalities. TC= total cholesterol; LDL-C = low density lipoprotein cholesterol; HDL-C = high density lipoprotein cholesterol; TG = triglycerides.

Dyslipidaemia was observed in 71% of the study participants (Figure 3). Notably, elevated LDL-C concentrations were the most frequent lipid abnormality observed in 55% of the participants, and the least frequent type of lipid abnormality was decreased levels of HDL-C.

Table 3 summarises the dyslipidaemia patterns regarding single, combined and mixed dyslipidaemia among participants who had dyslipidaemia, respectively. Isolated dyslipidaemia was the most common form, seconded by combined dyslipidaemia, and mixed dyslipidaemia was the least common pattern. In all the dyslipidaemia categories, elevated LDL-C was highly prevalent.

### Risk factors associated with dyslipidaemia among the study participants.

Dyslipidaemia was positively associated with female sex, overweight and obesity (Table 3). T2DM was not statistically significantly associated with dyslipidaemia. Smoking status, alcohol consumption, education, occupation and HBA1c greater than 7% showed no significant association with dyslipidaemia in this study population. Overweight and obesity emerged as the independent predictor of dyslipidaemia [AOR 1.8 ( 95% CI 1.15- 3.37); p= 0.04] after adjusting for sex, age ≥ 30 years, physical exercise, and HIV status. Table 3 summarises these findings.

## Discussion

This study at the largest tertiary referral hospital in central Malawi reports a high rate of dyslipidemia among adult individuals with diabetes, with elevated LDL-C as the most common lipid abnormality. Dyslipidaemia was positively associated with overweight and obesity, female sex, and age above 30 years. Overweight and obesity emerged as an independent predictor for dyslipidaemia among the study participants.

The study's high prevalence of dyslipidaemia is comparable to other studies among individuals with DM in other Sub-Saharan African countries (3, 10, 13). These results are consistent with the previously reported high prevalence of dyslipidaemia at another public tertiary referral hospital in Malawi among individuals with DM and hypertension (14). Dyslipidaemia is a significant modifiable determinant of ASCD globally and in Africa (5, 15) and requires proactive screening and management, especially among individuals at high risk, such as those living with DM. Sadly, there is a lack of aggressive efforts towards addressing dyslipidaemia among individuals with DM (14). Plausibly, the poor screening and management of dyslipidaemia contribute to the escalating rates of ASCDs in such LMICs (16).

Elevated LDL-C was the most common lipid abnormality in the present study. Elevated LDL-C-C drives atherosclerosis and is the target for treating dyslipidaemia (17, 18). Therefore, the study participants were at higher risk for ASCDs, given the elevated levels of LDL-C, and should be treated with lipid-lowering therapy (18). Ideally, all patients with DM aged between 40 and 75 years, regardless of the presence of complications, are recommended to be on statins for the primary prevention of ASCDs to achieve an LDL-C target of < 70mg/dl (18, 19). More than 80% of the study participants were between 40 and 75 years old and ideally required at least moderate-intensity statin therapy (18, 19). In the present study, we did not investigate the proportion of participants who were on lipid-lowering pharmacotherapy, as it was beyond the scope of this study. However, dyslipidaemia treatment rates among people with DM were reported at 0% at another public tertiary hospital in Malawi (14). Factors such as frequent stock-outs of lipogram reagents, unavailability of local guidelines for the management of dyslipidaemia and inexperience healthcare personnel hinder the efforts towards addressing dyslipidaemia among people with DM in Malawi and other LMICs (14, 20, 21). It is imperative, therefore, that the Ministry of Health in Malawi and its partners reinforce efforts toward strategies for optimally addressing dyslipidaemia among patients with DM to reduce the risk of ASCDs.

Overweight and obesity were highly prevalent (70%) in this study and independently predicted dyslipidaemia. The rates of overweight and obesity among the participants were higher than the 40% rate reported in the general population in urban Malawi (22). The difference may be because the present study involved participants with DM, of whom 81% had T2DM, for which overweight and obesity are known risk factors (23). However, given the study's cross-sectional nature, it was complex to establish the temporal relationship between DM, overweight and obesity and dyslipidaemia. Similar to our results, other African studies have reported overweight and obesity as independent predictors of dyslipidaemia (4, 6, 13). Socio-economic transitions such as urbanisation influence sedentary lifestyles and poor dietary habits, promoting overweight and obesity in Africa and Malawi (22, 24). Recent data suggest that more than two-thirds of the Malawi adult population do not consume recommended adequate amounts daily of fruits and vegetables (25). Adequate consumption of whole fruits and vegetables is essential for better BMI control and normal nutrition (26), and should be encouraged, especially among people with DM (26).

Females in this study were more likely to be overweight and obese than men, as reported in Malawi (22) and other African countries (23). Overweight and obesity in women in Malawi, like in many African countries, are influenced by the socio-cultural context where it is revered and regarded as a sign of high economic standing, beauty and a sign of fertility (23, 27, 28). Additionally, in urban settings, women are engaged mainly in jobs requiring less physical activity. In Lilongwe city, the risk of obesity is expectedly high (27, 29). Overweight and obesity must be addressed among individuals with DM to curb dyslipidaemia and the risk of ASCDs in this population (9). Malawi has in-country-trained dieticians working at tertiary-level healthcare facilities and should reinforce lifestyle modifications, including proactive physical activity and appropriate cardiovascular-friendly dietary habits, towards reducing the rates of overweight and obesity among individuals with DM (30, 31).

Poor glycaemic control, as indicated by HBA1c ≥ 7%, was observed in 85% of participants in the present study. These results are similar to previous studies in Southern Africa (10). If left unmanaged, poor glycaemic control positively influences dyslipidaemia (32) and increases the risk of cardiovascular events for every 1% increase in HBA1c level (33). These considerations necessitate re-strategising the monitoring and delivery of efficacious interventions like dietary adjustments, glucose monitoring and physical exercising to help improve glucose control.

Although smoking and alcohol consumption are known risk factors for dyslipidaemia, there was no statistically significant association with dyslipidaemia in this study. The prevalence of smoking and alcohol consumption in this study was lower than NCD STEPwise survey findings (1% and 5% versus 11.2% and 17%, respectively) (25). The lack of significant association would have likely been due to the lack of statistical power, owing to the low prevalence of the conditions in this study. Moreover, the participants may have underreported since they are advised to abstain from alcohol and smoking at the DM clinic during health education sessions.

The HIV prevalence in the study population was 11%. A previous study in Malawi showed that the rates may be as high as 20% among people with DM (14). Dyslipidaemia and DM increase the risk of ASCDs up to 2.4-fold in people living with HIV (PLWH) (19, 34-37). In this study, the association of HIV and dyslipidaemia approached significance, and low power may have influenced the lack of statistical significance due to the low prevalence of HIV among the participants. Nevertheless, it is essential to carefully consider the choice of lipid-lowering therapy in PLWH with DM and dyslipidaemia (38). Atorvastatin is the preferred choice of statin among PLWH, unlike simvastatin and rosuvastatin, due to the high risk of drug interactions with antiretroviral drugs (38, 39). Clinicians must evaluate potential drug-drug interactions between lipid-lowering agents and ARVs before prescription (38, 40).

This study had its limitations. The cross-sectional nature precluded any temporal association between the risk factors and dyslipidaemia. In addition, the molecular and humoral mechanisms, such as the influence of adiponectin in the pathophysiology of obesity and dyslipidaemia, were not evaluated, as these were beyond the scope of this study (41, 42). Whilst the study complements previous findings on diabetic dyslipidaemia in Malawi at tertiary hospitals, the results may not be generalizable to the rural settings of Malawi. Studies from rural settings would help inform country-wide clinical guidelines on screening and management of diabetic dyslipidaemia in Malawi.

## Conclusion

This study highlights the high prevalence of dyslipidaemia characterised by a high frequency of elevated LDL-C, with overweight and obesity as an independent positive predictor. The results underscore the importance of appropriately addressing dyslipidaemia, overweight and obesity among individuals with DM in Malawi and other LMICs as one of the significant ways of reducing the risk of ASCDs among individuals with DM. Lifestyle modifications such as better dietary habits, physical activity and pharmacotherapy should be reinforced for primary and secondary prevention of cardiovascular events. Data in rural settings of Malawi are required to provide generalised data on the magnitude of dyslipidaemia in patients with DM in Malawi. Additionally, the availability and impact of statin use in public hospitals in Malawian patients with DM warrant further research attention.

## Figures and Tables

**Table 1.** Participants' baseline characteristics.

Variable	n (%)	
**Age category**		
18-30	30 (8)	
31-40	43 (11)	
41-50	93 (24)	
More than 50	225 (58)	
**Gender**		
Male	141 (36)	
Female	250 (64)	
**Education level**		
None	23 (6)	
Primary	163 (42)	
Secondary	161 (41)	
Tertiary	44 (11)	
**Occupation**		
No formal employment	143 (36)	
Farmer	57 (15)	
Business	118 (30)	
Formal employment	73 (19)	
**Type of DM**		
Type 1		73 (19)
Type 2		318 (81)
**Smoking Status**		
Yes	4 (1)	
No	387 (99)	
**Alcohol Consumption**		
Yes	18 (5)	
No	373 (95)	
**Intentional exercise intensity**		
None	178 (46)	
Minimum 30 minutes/day	138 (35)	
More than 30 minutes/day	75 (19)	
**HIV Status**		
Reactive	42 (11)	
Non-Reactive	296 (76)	
Unknown	53 (13)	
**Duration since diagnosis**		
< 5 years	189 (49)	
5-10 years	125 (32)	
10.1-15 years	36 (9)	
>15 years	41(10)	
**High Blood Pressure Reading (140 mmHg systolic, mmHg diastolic)**		
No	174 (44)	
Yes	222 (56)	
**HBA1c >7%**		
No	58 (15)	
Yes	338 (85)	

**Table 3.** Dyslipidaemia pattern distribution among study participants who had dyslipidaemia (n=277)

Dyslipidaemia type	Parameter	N (%)
		
**Isolated dyslipidaemia**		**181 (65.34)**
	TC	7 (2.53)
	LDL	123 (44.40)
	TG	19 (6.86)
	HDL	32 (11.55)
**Combined dyslipidaemia**		**89 (32.13)**
	LDL+TG	70 (25.27)
	LDL+HDL	9 (3.25)
	HDL+TG	10 (3.61)
**Mixed dyslipidaemia**		**7 (2.53)**
	LDL+TG+HDL	7 (2.53)
**Total**		**277 (100)**

**Table 3.** Associated risk factors for dyslipidaemia in the study participants.

Variable	Reference	Crude OR (95% CI)	p- value	Adjusted OR (95% CI)	p- value
Female sex	Male sex	1.65 (1.05 - 2.58)	0.029*	0.74 (0.44 - 1.25)	0.25
Age ≥ 30 years	<30 years	3.60 (1.17 - 7.68)	0.001*	2.16 (0.92 - 5.08)	0.07
Diabetes type	Type 2	0.88 (0.39 - 1.14)	0.140	-	-
Alcohol consumption	No consumption	0.81 (0.30 - 2.20)	0.677	-	-
Physical exercise	Below WHO recommendation	0.50 (0.24 - 1.03)	0.061	0.46 (0.41-1.09)	0.06
HIV positive	HIV negative	1.84 (0.91 - 4.57)	0.07	2.11 (0.92-4.83)	0.08
Overweight & obese	Normal BMI	2.11 (1.33 - 3.34)	0.002*	1.80 (1.02- 3.19)	0.04*
HBA1c	Per 1% increase	1.00 (0.93 - 1.07)	0.972	-	-

## Abbreviations

ASCVD: Atherosclerotic Cardiovascular Disorder
BMI: Body Mass Index
CI: Confidence Interval
COMREC: College of Medicine Research and Ethics Committee
COVID-19: Coronavirus disease 2019
DM: DM Mellitus
EDTA: Ethylenediaminetetraacetic acid
GCP: Good Clinical Practice
HBA1c: Glycosylated haemoglobin
HDL-C: High-density lipoprotein cholesterol
HIV: Human immunodeficiency virus
KCH: Kamuzu Central Hospital
LDL-C: Low-density lipoprotein cholesterol
OR: Odds ratio
ARR: Adjusted odds ratio
T1DM: Type 1 Diabetes Mellitus
T2DM: Type 2 Diabetes Mellitus
TC: Total Cholesterol
TG: Triglycerides
VLDL: Very low-density lipoprotein
USA: United States of America
WHO: World Health Organisation

## References

[1] Federation ID. IDF Diabetes Atlas Brussels. Belgium: International Diabetes Federation; 2021.

[2] Knoema. Malawi - Diabetes prevalence as a share of population aged 20–79 years 2021 [updated 2023; cited 2023 February ]. Available from: https://knoema.com/atlas/Malawi/topics/Health/Nutrition/Diabetes-prevalence.

[3] Pitso L, Mofokeng TRP, Nel R. Dyslipidaemia pattern and prevalence among type 2 diabetes mellitus patients on lipid-lowering therapy at a tertiary hospital in central South Africa. BMC Endocr Disorders. 2021;21(1):1–10. 10.1186/s12902-021-00813-7 PMC8349492 34365977

[4] Noubiap JJ, Bigna JJ, Nansseu JR, Nyaga UF, Balti EV, Echouffo-Tcheugui JB, Prevalence of dyslipidaemia among adults in Africa: a systematic review and meta-analysis. The Lancet Global Health. 2018;6(9):e998–e1007. 30103999 10.1016/S2214-109X(18)30275-4

[5] Roth GA, Mensah GA, Johnson CO, Addolorato G, Ammirati E, Baddour LM, Global burden of cardiovascular diseases and risk factors, 1990–2019: update from the GBD 2019 study. J Am Coll Cardiol. 2020;76(25):2982–3021. 33309175 10.1016/j.jacc.2020.11.010 PMC7755038

[6] Lumu W, Kampiire L, Akabwai GP, Ssekitoleko R, Kiggundu DS, Kibirige D. Dyslipidaemia in a Black African diabetic population: burden, pattern and predictors. BMC Res Notes. 2017;10(1):1–7. 29121994 10.1186/s13104-017-2916-y PMC5679328

[7] Athyros VG, Doumas M, Imprialos KP, Stavropoulos K, Georgianou E, Katsimardou A, Diabetes and lipid metabolism. Hormones. 2018;17:61–7. 29858856 10.1007/s42000-018-0014-8

[8] Blom DJ. Dyslipidaemia in Type 2 diabetes. South Afr J Diabetes Vascular Disease. 2013;10(2):48–50.

[9] Schofield JD, Liu Y, Rao-Balakrishna P, Malik RA, Soran H. Diabetes dyslipidemia. Diabetes therapy. 2016;7:203–19. 27056202 10.1007/s13300-016-0167-x PMC4900977

[10] Daya R, Bayat Z, Raal F. Prevalence and pattern of dyslipidaemia in type 2 diabetes mellitus patients at a tertiary care hospital. J Endocrinol Metabolism Diabetes South Afr. 2017;22(3):31–5.

[11] Jellinger PS, Handelsman Y, Rosenblit PD, Bloomgarden ZT, Fonseca VA, Garber AJ, American Association of Clinical Endocrinologists and American College of Endocrinology guidelines for management of dyslipidemia and prevention of cardiovascular disease. Endocr Pract. 2017;23:1–87. 10.4158/EP171764.APPGL 28437620

[12] Organization WH. Obesity: preventing and managing the global epidemic. 2000. 11234459

[13] Obsa MS, Ataro G, Awoke N, Jemal B, Tilahun T, Ayalew N, Determinants of Dyslipidemia in Africa: a systematic review and meta-analysis. Front Cardiovasc Med. 2022;8:2249. 10.3389/fcvm.2021.778891 PMC8904727 35284497

[14] Katundu KG, Mukhula V, Phiri T, Phiri C, Filisa-Kaphamtengo F, Chipewa P, High prevalence of dyslipidaemia among persons with diabetes mellitus and hypertension at a tertiary hospital in Blantyre, Malawi. BMC Cardiovasc Disord. 2022;22(1):1–11. 36544081 10.1186/s12872-022-03011-y PMC9771776

[15] Owolabi MO, Sarfo F, Akinyemi R, Gebregziabher M, Akpa O, Akpalu A, Dominant modifiable risk factors for stroke in Ghana and Nigeria (SIREN): a case-control study. The Lancet Global Health. 2018;6(4):e436–e46. 29496511 10.1016/S2214-109X(18)30002-0 PMC5906101

[16] Capewell S, Ford ES. Why have total cholesterol levels declined in most developed countries? BMC Public Health. 2011;11:1–5. 21834954 10.1186/1471-2458-11-641 PMC3199603

[17] Ference BA, Ginsberg HN, Graham I, Ray KK, Packard CJ, Bruckert E, Low-density lipoproteins cause atherosclerotic cardiovascular disease. 1. Evidence from genetic, epidemiologic, and clinical studies. A consensus statement from the European Atherosclerosis Society Consensus Panel. Eur Heart J. 2017;38(32):2459–72. 28444290 10.1093/eurheartj/ehx144 PMC5837225

[18] Michos ED, McEvoy JW, Blumenthal RS. Lipid management for the prevention of atherosclerotic cardiovascular disease. N Engl J Med. 2019;381(16):1557–67. 31618541 10.1056/NEJMra1806939

[19] Grundy SM, Stone NJ, Bailey AL, Beam C, Birtcher KK, Blumenthal RS, AHA/ACC/AACVPR/AAPA/ABC/ACPM/ADA/AGS/APhA /ASPC/NLA/PCNA guideline on the management of blood cholesterol: a report of the American College of Cardiology/American Heart Association Task Force on Clinical Practice Guidelines. Journal of the American College of Cardiology. 2019;73(24):e285–e350. 30423393 10.1016/j.jacc.2018.11.003

[20] Jingi AM, Nansseu JRN, Noubiap JJNJBed. Primary care physicians’ practice regarding diabetes mellitus diagnosis, evaluation and management in the West region of Cameroon. 2015;15(1):18. 10.1186/s12902-015-0016-3 PMC4403824 25881080

[21] Moyo K, Porter C, Kabue M, Chilima B, Zungu L, Mwenda R, Use of laboratory test results in patient management by clinicians in Malawi. Afr J Lab Med. 2015;4(1):1–8. 10.4102/ajlm.v4i1.277 PMC4870597 27213139

[22] Price AJ, Crampin AC, Amberbir A, Kayuni-Chihana N, Musicha C, Tafatatha T, Prevalence of obesity, hypertension, and diabetes, and cascade of care in sub-Saharan Africa: a cross-sectional, population-based study in rural and urban Malawi. The lancet Diabetes & endocrinology. 2018;6(3):208–22. 29371076 10.1016/S2213-8587(17)30432-1 PMC5835666

[23] Issaka A, Paradies Y, Stevenson C. Modifiable and emerging risk factors for type 2 diabetes in Africa: a systematic review and meta-analysis protocol. Syst reviews. 2018;7(1):1–10. 10.1186/s13643-018-0801-y PMC6136189 30208942

[24] Peer N, Steyn K, Lombard C, Gwebushe N, Levitt N. A high burden of hypertension in the urban black population of Cape Town: The Cardiovascular Risk in Black South Africans (CRIBSA) Study. PLoS ONE. 2013;8(11):e78567. 24250798 10.1371/journal.pone.0078567 PMC3826752

[25] MINISTRY OF HEALTH and Population Malawi. MALAWI NATIONAL STEPwise SURVEY FOR NON-COMMUNICABLE DISEASES RISK FACTORS 2017 REPORT. Lilongwe 2020.

[26] Yen TS, Htet MK, Lukito W, Bardosono S, Setiabudy R, Basuki ES, Increased vegetable intake improves glycaemic control in adults with type 2 diabetes mellitus: a clustered randomised clinical trial among Indonesian white-collar workers. J Nutritional Sci. 2022;11:e49. 10.1017/jns.2022.41 PMC9241062 35836691

[27] Kanter R, Caballero B. Global gender disparities in obesity: a review. Adv Nutr. 2012;3(4):491–8. 22797984 10.3945/an.112.002063 PMC3649717

[28] Steyn NP, Mchiza ZJ. Obesity and the nutrition transition in Sub-Saharan Africa. Ann N Y Acad Sci. 2014;1311(1):88–101. 24725148 10.1111/nyas.12433

[29] Malawi Government. 2018 Malawi Population and Housing Census report,. In: Office NS, editor. 2019.

[30] Arnett D, Blumenthal R, Albert M, i wsp, ACC/AHA Guideline on the Primary Prevention of Cardiovascular Disease. 2019 : Executive Summary: A Report of the American College of Cardiology/American Heart Association Task Force on Clinical Practice Guidelines. J Am Coll Cardiol. 2019;74(10):1376 – 414. 30894319 10.1016/j.jacc.2019.03.009 PMC8344373

[31] Katundu K. An observational study of perioperative nutrition and postoperative outcomes in patients undergoing laparotomy at Queen Elizabeth Central Hospital in Blantyre, Malawi. Malawi Med J. 2018;30(2):79–85. 30627333 10.4314/mmj.v30i2.5 PMC6307065

[32] Mahajan R, Koley S. Association of HbA1c with Lipid profiles in patients with type 2 diabetes mellitus. Int J Biomed Res. 2016;7(3):139–43.

[33] Poreba M, Rostoff P, Siniarski A, Mostowik M, Golebiowska-Wiatrak R, Nessler J, Relationship between polyunsaturated fatty acid composition in serum phospholipids, systemic low-grade inflammation, and glycemic control in patients with type 2 diabetes and atherosclerotic cardiovascular disease. Cardiovasc Diabetol. 2018;17(1):1–11. 29452596 10.1186/s12933-018-0672-5 PMC5815243

[34] Association AD. 10. Cardiovascular disease and risk management: Standards of Medical Care in Diabetes—2019. Diabetes Care. 2019;42(Supplement 1):S103–S23. 30559236 10.2337/dc19-S010

[35] Subramanian S, Chait A. Dyslipidemia in diabetes. 2019.

[36] Feinstein MJ, Hsue PY, Benjamin LA, Bloomfield GS, Currier JS, Freiberg MS, Characteristics, prevention, and management of cardiovascular disease in people living with HIV: a scientific statement from the American Heart Association. Circulation. 2019;140(2):e98–e124. 31154814 10.1161/CIR.0000000000000695 PMC7993364

[37] Durand M, Sheehy O, Baril J-G, Lelorier J, Tremblay CL. Association between HIV infection, antiretroviral therapy, and risk of acute myocardial infarction: a cohort and nested case–control study using Quebec's public health insurance database. JAIDS J Acquir Immune Defic Syndr. 2011;57(3):245–53. 21499115 10.1097/QAI.0b013e31821d33a5

[38] Klug E, Raal F, Marais A, Smuts C, Schamroth C, Jankelow D, South African dyslipidaemia guideline consensus statement: 2018 update A joint statement from the South African Heart Association (SA Heart) and the Lipid and Atherosclerosis Society of Southern Africa (LASSA). South Afr Med J. 2018;108(11b):973–1000. 30421699

[39] Meintjes G, Moorhouse MA, Carmona S, Davies N, Dlamini S, Van Vuuren C, Adult antiretroviral therapy guidelines 2017. South Afr J HIV Med. 2017;18(1):1–24. 10.4102/sajhivmed.v18i1.776 PMC5843236 29568644

[40] Liverpool Uo. HIV Drug Interactions [Available from: https://www.hiv-druginteractions.org/.

[41] Ghoshal K, Chatterjee T, Chowdhury S, Sengupta S, Bhattacharyya M. Adiponectin genetic variant and expression coupled with lipid peroxidation reveal new signatures in diabetic dyslipidemia. Biochem Genet. 2021;59:781–98. 33543406 10.1007/s10528-021-10030-5

[42] Yamamoto S, Matsushita Y, Nakagawa T, Hayashi T, Noda M, Mizoue T. Circulating adiponectin levels and risk of type 2 diabetes in the Japanese. Nutr diabetes. 2014;4(8):e130–e. 25133442 10.1038/nutd.2014.27 PMC4151175

